# Chromosome-scale genome assembly of the high royal jelly-producing honeybees

**DOI:** 10.1038/s41597-021-01091-7

**Published:** 2021-11-25

**Authors:** Lianfei Cao, Xiaomeng Zhao, Yanping Chen, Cheng Sun

**Affiliations:** 1 Institute of Animal Husbandry and Veterinary Science, Zhejiang Academy of Agricultural Sciences, Hangzhou, 310021 China; 2 Institute of Apicultural Research, Chinese Academy of Agricultural Sciences, Beijing, 100093 China; 3 USDA-ARS Bee Research Laboratory, USDA-ARS, Bldg. 306, BARC-East, Beltsville, MD 20705 USA

**Keywords:** Genetics, Agriculture

## Abstract

A high royal jelly-producing strain of honeybees (HRJHB) has been obtained by successive artificial selection of Italian honeybees (*Apis mellifera ligustica*) in China. The HRJHB can produce amounts of royal jelly that are dozens of times greater than their original counterparts, which has promoted China to be the largest producer of royal jelly in the world. In this study, we generated a chromosome-scale of the genome sequence for the HRJHB using PacBio long reads and Hi-C technique. The genome consists of 16 pseudo-chromosomes that contain 222 Mb of sequence, with a scaffold N50 of 13.6 Mb. BUSCO analysis yielded a completeness score of 99.3%. The genome has 12,288 predicted protein-coding genes and a rate of 8.11% of repetitive sequences. One chromosome inversion was identified between the HRJHB and the closely related Italian honeybees through whole-genome alignment analysis. The HRJHB’s genome sequence will be an important resource for understanding the genetic basis of high levels of royal jelly production, which may also shed light on the evolution of domesticated insects.

## Abstract (summary)

**Table:** 

Measurement(s)	genome • DNA • transcriptome • sequence_assembly
Technology Type(s)	DNA sequencing • RNA sequencing • sequence assembly process
Sample Characteristic - Organism	Apis mellifera

Machine-accessible metadata file describing the reported data: 10.6084/m9.figshare.16879888

## Background & Summary

**Fig. 1:**
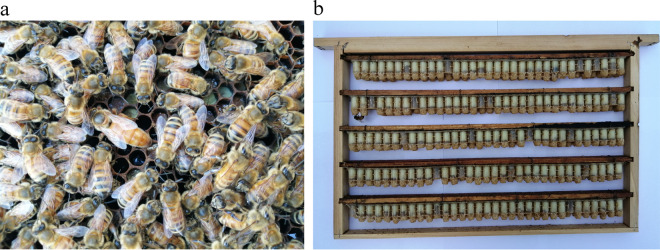
High royal jelly-producing honeybees (HRJHB) in China. (**a**) Queen and workers in one colony. (**b**) Royal jelly in the queen’s cells.

Royal jelly (RJ) is a proteinaceous secretion synthesized by the hypopharyngeal and mandibular glands of nurse worker bees and is used for feeding queen and larvae^1^. It also plays a critical role in the caste determination of honeybees^2^. Nowadays, RJ is widely used in medical products, health foods and cosmetics in many countries owing to the numerous biological activities it is known to perform including anti-bacterial, anti-oxidative, anti-inflammatory, immunomodulatory, anti-tumoral, and anti-aging activities^3,4^. China is now the largest producer and exporter of RJ in the world, which satisfies nearly all the global demand^5^. Since the 1980s, the yearly production of RJ in China has increased from 200 to around 3000 tons^5^. The rapidly increased production of RJ in China has been mainly attributed to the successful breeding of the high royal jelly-producing honeybees (HRJHB) (Fig. 1), and the effective utilization of corresponding production tools and techniques^6^.

HRJHB was derived from an Italian honeybee subspecies (*Apis mellifera ligustica*), which was chiefly introduced into China in the 1910s–1930s^7^. In 1960s, attempts were made by beekeepers in the Southeast region of China to select high RJ producing bee stocks to meet a high demand for RJ^8^. The colony that displayed a high rate of RJ production was selected for raising daughter queens and drones in each apiary^8^. Sometimes queens were also developed using larvae of high RJ producing colonies from different apiaries^8^. Queens then open-mated with local drones in the air^8^. After the aforementioned semi-controlled style of breeding, the annual RJ production per colony increased from 0.2–0.3 kg in the 1960s to 2–3 kg in the late 1980s and even reaching 6–8 kg in the 2000s^8^. This was perceived as a miracle and the HRJHB was rapidly introduced to other regions of China from the 1980s, onwards as well as other countries at a later date. At present, the annual production per HRJHB colony has reached more than 10 kg, which is dozens of times greater than that of common Italian honeybees (*A. m. ligustica*)^5^. RJ production has become a major income source for many beekeepers in China and the HRJHB has been certified as a new honeybee genetic resource by the Chinese government^7^.

Previous studies regarding isoenzymes, microsatellites and mitochondrial DNA have shown significant genetic differentiation between the HRJHB and the other common *A. m. ligustica* populations in China^9–11^. It was suggested that morphological markers, behavioural and physiological changes, and differently expressed proteins and genes, correlate to the high royal jelly-producing trait^12–16^. However, related research has so failed to develop an entirely clear picture of what causes the complex royal jelly-producing trait. In recent years, honeybee selection programs for high RJ production have also been implemented in Brazil and France beekeeping^17,18^. Additionally, further breeding of HRJHB for improving general resistance to disease is being carried out in China.

**Fig. 2:**
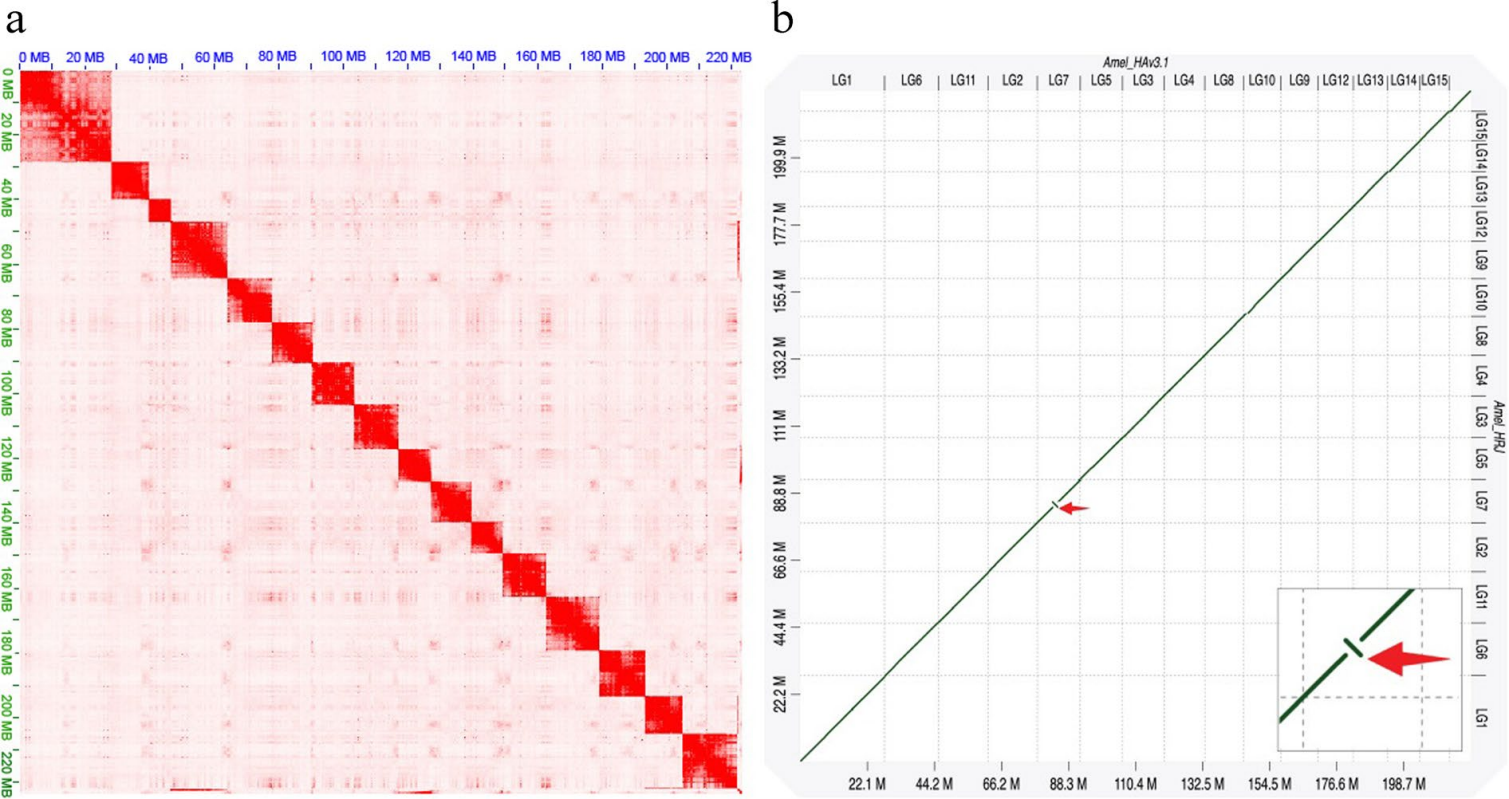
Chromosome-scale assembly for HRJHB genome. (**a**) The HRJHB’s genome contig contact matrix using Hi-C data. (**b**) The HRJHB’s genome sequence was aligned with a closely related honeybee genome (NCBI assembly: Amel_HAv3). The red arrow indicates the chromosome inversion between the two genomes on LG7.

In this study, we generated a chromosome-scale of the genome assembly of the HRJHB using PacBio long-reads, Illumina short reads, and the Hi-C chromosome conformation capture technique (Table 1; Fig. 2a). The resultant genome has a total length of 222 Mb with 16 chromosomes, and the scaffold N50 was 13.6 Mb (Table 1). One chromosome inversion was identified between HRJHB and the closely related Italian honeybees via whole-genome alignment analysis (Fig. 2b). Moreover, through a combination of *ab initio* gene predictions, transcript evidence and homologous protein evidence, 12,288 protein coding genes were identified in this genome, therein 6,615 genes were assigned a GO term and 8,614 genes were assigned a protein domain (Table 2). Repetitive elements are made of 8.11% of the HRJHB genome sequence, but transposable elements (TEs) only occupy 2.15% (Table 2). Among those TEs, DNA transposons represented the most abundant TE class, which make up the majority of the total TE content (1.68% out of 2.15%). Furthermore, *Tc1*-mariner (TcMar) is the most abundant TE superfamily in the genome. The genome sequence provides a valuable resource for exploring the molecular basis of the high royal jelly-producing trait in honeybee and will facilitate further genetic improvements. The HRJHB may even represent a novel animal model for studying the effects of artificial selection on insects.

**Table 1:** Sequencing data generated for the HRJHB genome assembly.

Genome sequencing			
	Read number	Read_length(mean)	Total read length (Gb)
PacBio long reads	2,154,163	15,489	33.37
Ilumina sequencing	71786450	150	10.77
RNA-seq	130258000	150	18.05
Hi-C sequencing	218592996	150	32.79
**Genome assembly**			
Genome assembly size	222 Mb		
Number of scaffolds	16		
Scaffold N50	13.6 Mb		
BUSCO completeness	99.30%

**Table 2:** Annotation of protein-coding genes and repetitive sequences.

**Protein-coding genes**			
	Total gene number	12,288	
	BUSCO completeness	97%	
	Number of genes with a GO term	6,615	
	Number of genes with a protein domain	8,614	
**Repetitive sequences**			
	**TE superfamily**	**Length occupied (bp)**	**Percent of genome**
DNA transposons	TcMar	3557056	1.67
	hAT	14338	0.01
non-LTR retrotransposons	CR1	952652	0.45
	R2	49376	0.02
LTR retrotransposons	Copia	6014	0.00
	Gypsy	383280	0.18
Total TEs		4962716	2.15
Other repeats		13761592	5.96
Total repeats		18724308	8.11

## Methods

### Sample collection and genome sequencing

**Fig. 3:**
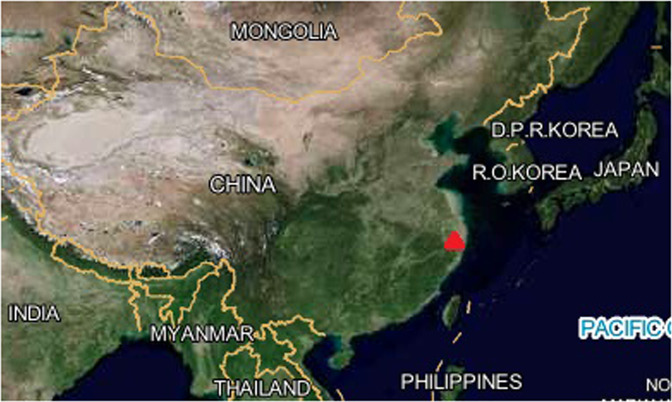
Original area of HRJHB (red arrowhead).

Samples of the HRJHB for genome and transcriptome sequencing were collected in 2019 from Zhejiang Province, China, where the HRJHB was originated and primarily distributed (Fig. 3).

Newly emerged drone bees (n = 20), that are descendants of the queen bee, were collected from a single colony (Fig. 1a). The thoraxes were pooled for PacBio single molecule real-time (SMRT) sequencing and Illumina HiSeq sequencing. Genomic DNA was extracted using the Gentra Puregene Tissue Kit (Qiagen) and was sequenced in accordance with the standard protocols. Newly emerged worker bees (n = 20) were collected from the same colony and their thoraxes were pooled for Hi-C sequencing. Hi-C library preparation was performed by Frasergen (http://www.frasergen.com/), which mainly followed a protocol described previously^19^. The obtained Hi-C sequencing libraries were sequenced on the Illumina HiSeq X Ten platform. Worker bees that were excreting royal jelly (n = 20) were collected from the same colony and their heads, thoraxes and abdomens (excluding the mid-gut tissues) were pooled for RNA-seq on the Illumina HiSeq X Ten platform.

### *De novo* genome assembly for HRJHB

A total of 33.37 Gb of long reads were generated by the PacBio Sequel platform (Table 1), which were self-corrected and assembled into contigs using Canu v2.1^20^, with default parameters. The obtained contigs were parsed by Purge Haplotigs v1.1.1^21^ to get rid of the redundancies caused by the heterozygosity of the pooled honeybee samples. Then, the remaining non-redundant contigs were polished with Illumina HiSeq reads (Table 1) three times by utilizing software Pilon v1.23^22^. Finally, the Juicer tool^23^ was applied to map Hi-C reads (Table 1) against the polished contig sequences of HRJHB using the BWA algorithm^24^. The 3D-DNA pipeline^25^ was applied to scaffold the contig sequences in relation to the chromosome-scale of genome assembly.

### Annotation of repeat sequences

TEs were *de novo* identified by RepeatModeler2^26^, in line with default parameters. Using the obtained repeat library, each honeybee genome assembly was analyzed with RepeatMasker (http://www.repeatmasker.org) to yield a comprehensive summary of the TE landscape in each assembly. The annotation files produced by RepeatMasker were processed by in-house scripts to eliminate redundancies. In addition, refined annotation files were used to determine the TE diversity and abundance within each assembly and tandem repeats were identified with the Tandem Repeat Finder^27^, which was implemented in RepeatMasker.

### Prediction and functional annotation of protein-coding genes

Annotation of protein-coding genes was based on *ab initio* gene predictions, transcript evidence, and homologous protein evidence, which were all applied in the MAKER computational pipeline^28^. Meanwhile, RNA-seq reads obtained in this study were assembled using Trinity^29^. The assembled RNA-seq transcripts, along with proteins from bees (superfamily Apoidea) that are available in the National Center for Biotechnology Information (NCBI) GenBank (last accessed on 01/28/2020), were imported into the MAKER pipeline to generate gene models. To obtain functional clues for the predicted gene models, protein sequences encoded by them were searched against the Uniprot-Swiss-Prot protein databases (last accessed on 01/28/2020) using the BLASTp algorithm implemented in BLAST suite v2.28^30^. In addition, protein domains and GO terms associated with gene models were identified by InterproScan-5^31^.

## Data Records

The raw data was submitted to the National Center for Biotechnology Information (NCBI) SRA database (Experiments for SRP300170) under BioProject accession number PRJNA689474^32^. The assembled genome has been deposited at DDBJ/ENA/GenBank under the accession GCA_019321825.1^33^. Moreover, the genome annotation results have been deposited at the Figshare database^34^.

## Technical Validation

### Evaluation of the genome assembly

The completeness of the genome assembly was evaluated using a set of 4,415 hymenopteran benchmarking universal single-copy orthologs (BUSCOs) using software BUSCO v3^35^. The results indicated that 99.3% of these BUSCOs were present in the genome assembly (Table 1), suggesting a remarkably complete assembly of the HRJHB genome.

**Fig. 4:**
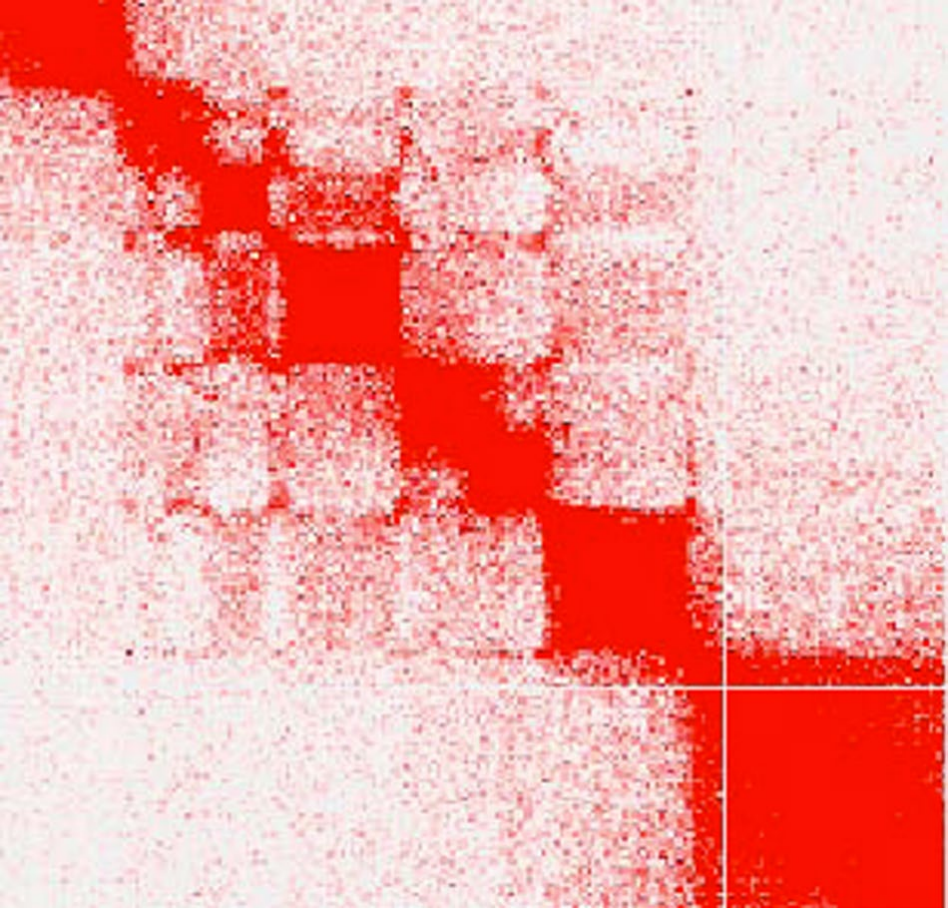
Hi-C heatmap around the identified chromosome inversion region in the HRJHB.

Furthermore, the chromosome-level structural accuracy was assessed by performing whole-genome alignments between HRJHB genome and a closely related honeybee genome (GenBank assembly: Amel_HAv3) using software D-GENIES^36^. The alignment results revealed a highly conserved chromosome structure between the two genomes, indicating an accurate scaffolding of contigs in the HRJHB genome. Nevertheless, we did find one inversion on LG7 (Fig. 2b). The Hi-C heatmap revealed a well-organized interaction contact pattern along the diagonals within/around the chromosome inversion region in HRJHB (Fig. 4), which rules out the possibility that the structural variation was derived from unreliable Hi-C signals in the HRJHB assembly. In addition, as chromosome inversion has been found to be associated with honeybee adaptations^37^, the inversion identified in the HRJHB genome will guarantee that further analysis will be carried out to investigate its association with high royal jelly production.

## Data Availability

All software used in this work is in the public domain, with parameters being clearly described in Methods. If no detail parameters were mentioned for a software, default parameters were used as suggested by developer.
